# Supplementary material for: Applicability of a Textile ECG-Belt for Unattended Sleep Apnoea Monitoring in a Home Setting
Source: Sensors (Basel). 2019 Jul 31;19(15):3367. doi: 10.3390/s19153367 (PMC6696177; doi:10.3390/s19153367)
Supplement: Supplementary file 1 [file sensors-19-03367-s001.zip › Supplementary2.docx]

Table S1. Artefact percentages and Poincaré standard deviations (n=12).

| **Subject** | **Hospital** | **Nights at home** | **Home** | **Poincaré** | |  |
| --- | --- | --- | --- | --- | --- | --- |
|  |  |  |  | **SD1** | **SD2** | |
| **1** | 5.4% | 1 | 1.1% | 38.99 | 186.43 | |
| **2** | 0.3% | 1 | 11.3% | 35.16 | 68.63 | |
|  |  | 2 | 6.3% | 33.73 | 107.19 | |
|  |  | 3 | 3.8% | 27.75 | 104.17 | |
| **3** | 2.5% | 1 | 2.6% | 28.03 | 168.91 | |
|  |  | 2 | 0.9% | 17.24 | 127.42 | |
|  |  | 3 | 1.3% | 20.79 | 145.58 | |
| **4** | 0.5% | 1 | 0.2% | 20.47 | 98.17 | |
|  |  | 2 | 0.3% | 18.99 | 101.07 | |
|  |  | 3 | 0.3% | 14.14 | 98.68 | |
| **5** | 4.8% | 1 | 1.3% | 34.39 | 157.90 | |
| **6** | 0.6% | 1 | 39.6% | 86.10 | 261.53 | |
|  |  | 2 | 24.6% | 75.99 | 175.70 | |
| **7** | 52.4% | 1 | 0.2% | 17.62 | 97.48 | |
|  |  | 2 | 0.3% | 15.35 | 94.96 | |
|  |  | 3 | 0.2% | 17.15 | 86.99 | |
| **8** | 9.1% | 1 | 0.6% | 11.39 | 74.53 | |
|  |  | 2 | 6.1% | 28.62 | 79.66 | |
|  |  | 3 | 5.4% | 23.02 | 93.81 | |
| **9** | 6.3% | 1 | 0.6% | 27.02 | 176.88 | |
|  |  | 2 | 0.6% | 26.47 | 181.57 | |
|  |  | 3 | 0.9% | 31.51 | 152.94 | |
| **10** | 16.5% | 1 | 12.1% | 80.76 | 183.26 | |
| **11** | 5.4% | 1 | 7.2% | 43.12 | 116.96 | |
|  |  | 2 | 6.2% | 33.68 | 119.51 | |
|  |  | 3 | 10.4% | 32.90 | 127.20 | |
| **12** | 11.1% | 1 | 15.1% | 53.67 | 192.77 | |
|  |  | 2 | 15.2% | 66.35 | 138.40 | |
