# Supplementary material for: Applicability of a Textile ECG-Belt for Unattended Sleep Apnoea Monitoring in a Home Setting
Source: Sensors (Basel). 2019 Jul 31;19(15):3367. doi: 10.3390/s19153367 (PMC6696177; doi:10.3390/s19153367)
Supplement: Supplementary file 1 [file sensors-19-03367-s001.zip › Supplementary3.docx]

Table S3: Overnight mean RR-Intervals (ms), unfiltered and filtered

|  |  | **Unfiltered** | | | | **filtered** | | | |
| --- | --- | --- | --- | --- | --- | --- | --- | --- | --- |
| **Subject** | **Nights** | **Gel electrode** | **ECG-belt clinics** | **ECG-belt home (mean)** | **ECG belt home (single)** | **Gel electrode** | **ECG-belt clinics** | **ECG-belt home (mean)** | **ECG belt home (single)** |
| **1** | 1 | 1078.7 | 1078.5 | 1025.6 | 1025.6 | 1081.2 | 1081.1 | 1023.4 | 1023.4 |
| **2** | 1 | 938.3 | 938.2 | 1096.2 | 996.1 | 935.8 | 936.0 | 950.7 | 997.4 |
|  | 2 |  |  |  | 1374.0 |  |  |  | 943.9 |
|  | 3 |  |  |  | 918.4 |  |  |  | 910.7 |
| **3** | 1 | 949.8 | 960.0 | 933.8 | 967.7 | 945.3 | 946.5 | 918.2 | 945.1 |
|  | 2 |  |  |  | 909.2 |  |  |  | 896.8 |
|  | 3 |  |  |  | 924.6 |  |  |  | 912.8 |
| **4** | 1 | 846.7 | 846.7 | 865.6 | 893.6 | 841.3 | 841.6 | 859.5 | 875.2 |
|  | 2 |  |  |  | 878.6 |  |  |  | 880.3 |
|  | 3 |  |  |  | 824.6 |  |  |  | 822.8 |
| **5** | 1 | 1133.4 | 1148.7 | 1011.4 | 1011.4 | 1124.5 | 1136.9 | 1016.5 | 1016.5 |
| **6** | 1 | 862.2 | 862.9 | 941.3 | 941.4 | 851.1 | 851.7 | 906.7 | 930.1 |
|  | 2 |  |  |  | 941.1 |  |  |  | 883.4 |
| **7** | 1 | 848.5 | 1221.2 | 897.0 | 887.6 | 843.7 | 926.2 | 885.1 | 878.3 |
|  | 2 |  |  |  | 888.1 |  |  |  | 886.5 |
|  | 3 |  |  |  | 915.5 |  |  |  | 890.6 |
| **8** | 1 | 881.1 | 921.3 | 972.1 | 859.5 | 859.1 | 867.4 | 856.4 | 856.5 |
|  | 2 |  |  |  | 994.0 |  |  |  | 845.8 |
|  | 3 |  |  |  | 1062.9 |  |  |  | 866.9 |
| **9** | 1 | 1184.5 | 1198.2 | 1167.2 | 1146.2 | 1180.9 | 1182.7 | 1152.0 | 1151.7 |
|  | 2 |  |  |  | 1149.4 |  |  |  | 1109.7 |
|  | 3 |  |  |  | 1206.1 |  |  |  | 1194.6 |
| **10** | 1 | 1083.9 | 1092.1 | 1130.1 | 1130.1 | 1035.9 | 1037.6 | 1064.8 | 1064.8 |
| **11** | 1 | 943.8 | 950.2 | 825.4 | 723.8 | 932.9 | 940.3 | 775.3 | 670.7 |
|  | 2 |  |  |  | 886.1 |  |  |  | 855.0 |
|  | 3 |  |  |  | 866.2 |  |  |  | 800.1 |
| **12** | 1 | 1114.3 | 1121.7 | 1135.0 | 1050.7 | 1099.3 | 1099.9 | 930.1 | 913.8 |
|  | 2 |  |  |  | 1219.4 |  |  |  | 946.4 |
