# Supplementary material for: Applicability of a Textile ECG-Belt for Unattended Sleep Apnoea Monitoring in a Home Setting
Source: Sensors (Basel). 2019 Jul 31;19(15):3367. doi: 10.3390/s19153367 (PMC6696177; doi:10.3390/s19153367)
Supplement: Supplementary file 1 [file sensors-19-03367-s001.zip › Supplementary4.docx]

Table S4. Overnight RR-intervall variability (SDNN), filtered and unfiltered

|  |  | **Unfiltered** | | | | **filtered** | | | |
| --- | --- | --- | --- | --- | --- | --- | --- | --- | --- |
| **Subject** | **Nights at home** | **Gel electrode** | **ECG-belt clinics** | **ECG-belt home (mean)** | **ECG belt home (single)** | **Gel electrode** | **ECG-belt clinics** | **ECG-belt home (mean)** | **ECG belt home (single)** |
| **1** | 1 | 145.4 | 145.4 | 77.1 | 77.1 | 103.1 | 103.0 | 72.5 | 72.5 |
| **2** | 1 | 88.8 | 89.8 | 1024.3 | 163.4 | 45.8 | 45.5 | 39.7 | 31.0 |
|  | 2 |  |  |  | 2776.7 |  |  |  | 42.7 |
|  | 3 |  |  |  | 132.8 |  |  |  | 45.4 |
| **3** | 1 | 113.0 | 125.3 | 136.1 | 134.0 | 58.4 | 60.9 | 58.0 | 64.7 |
|  | 2 |  |  |  | 61.8 |  |  |  | 53.1 |
|  | 3 |  |  |  | 212.4 |  |  |  | 56.1 |
| **4** | 1 | 133.9 | 133.1 | 70.2 | 106.8 | 45.5 | 45.2 | 47.8 | 48.0 |
|  | 2 |  |  |  | 55.1 |  |  |  | 51.1 |
|  | 3 |  |  |  | 48.6 |  |  |  | 44.4 |
| **5** | 1 | 324.3 | 351.3 | 93.7 | 93.7 | 108.9 | 96.6 | 87.6 | 87.6 |
| **6** | 1 | 217.2 | 223.8 | 277.2 | 316.5 | 34.2 | 33.5 | 91.7 | 120.6 |
|  | 2 |  |  |  | 238.0 |  |  |  | 62.8 |
| **7** | 1 | 74.2 | 504.9 | 57.7 | 56.4 | 37.1 | 160.9 | 36.4 | 35.0 |
|  | 2 |  |  |  | 41.1 |  |  |  | 36.2 |
|  | 3 |  |  |  | 75.6 |  |  |  | 37.9 |
| **8** | 1 | 253.4 | 316.3 | 880.9 | 49.7 | 32.4 | 42.5 | 39.3 | 37.1 |
|  | 2 |  |  |  | 1484.2 |  |  |  | 39.7 |
|  | 3 |  |  |  | 1108.8 |  |  |  | 41.3 |
| **9** | 1 | 128.1 | 183.7 | 166.1 | 78.2 | 78.3 | 78.9 | 76.8 | 74.4 |
|  | 2 |  |  |  | 284.5 |  |  |  | 70.4 |
|  | 3 |  |  |  | 135.6 |  |  |  | 85.7 |
| **10** | 1 | 194.0 | 217.1 | 247.6 | 247.6 | 109.4 | 110.0 | 117.1 | 117.1 |
| **11** | 1 | 150.0 | 154.0 | 153.3 | 119.6 | 81.1 | 64.0 | 53.3 | 39.2 |
|  | 2 |  |  |  | 143.3 |  |  |  | 62.2 |
|  | 3 |  |  |  | 196.9 |  |  |  | 58.7 |
| **12** | 1 | 267.9 | 326.2 | 906.0 | 868.3 | 73.4 | 75.6 | 66.9 | 68.1 |
|  | 2 |  |  |  | 943.8 |  |  |  | 65.7 |
