# Supplementary figures and images for: Applicability of a Textile ECG-Belt for Unattended Sleep Apnoea Monitoring in a Home Setting
Source: Sensors (Basel). 2019 Jul 31;19(15):3367. doi: 10.3390/s19153367 (PMC6696177; doi:10.3390/s19153367)

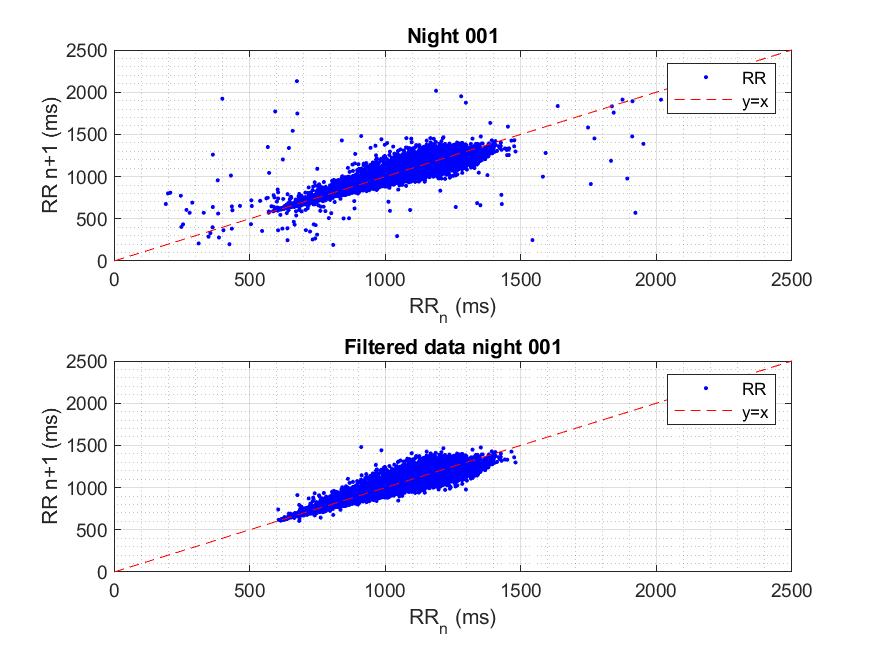

Supplement: Supplementary file 1 [file sensors-19-03367-s001.zip › Supplementary1/Night001_crr.jpg]

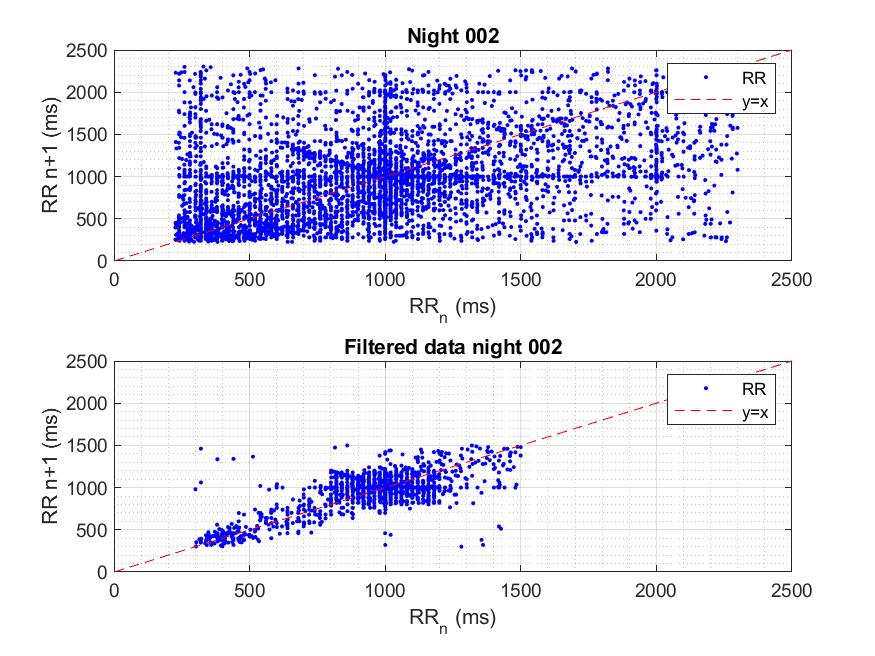

Supplement: Supplementary file 1 [file sensors-19-03367-s001.zip › Supplementary1/Night002_crr.jpg]

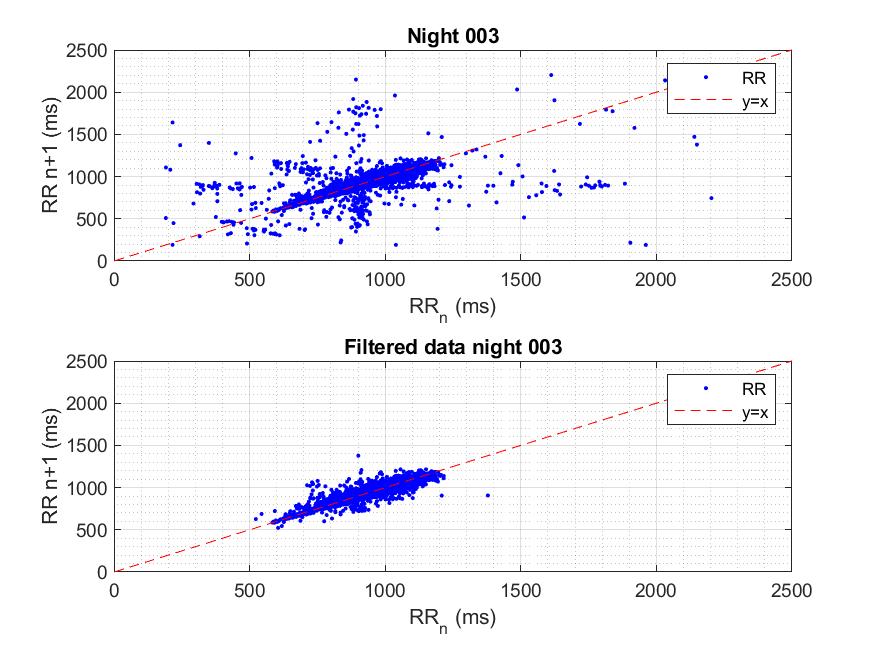

Supplement: Supplementary file 1 [file sensors-19-03367-s001.zip › Supplementary1/Night003_crr.jpg]

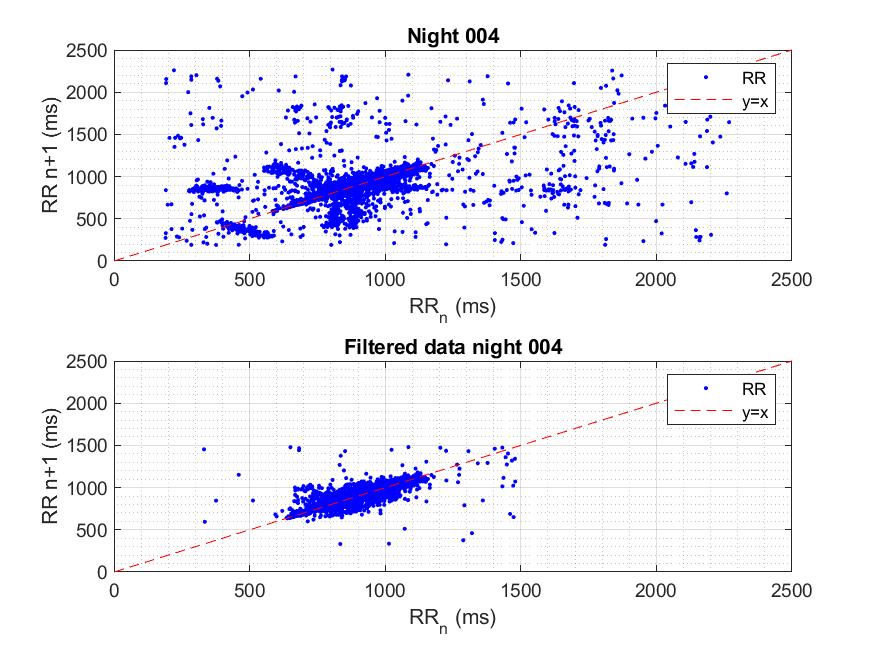

Supplement: Supplementary file 1 [file sensors-19-03367-s001.zip › Supplementary1/Night004_crr.jpg]

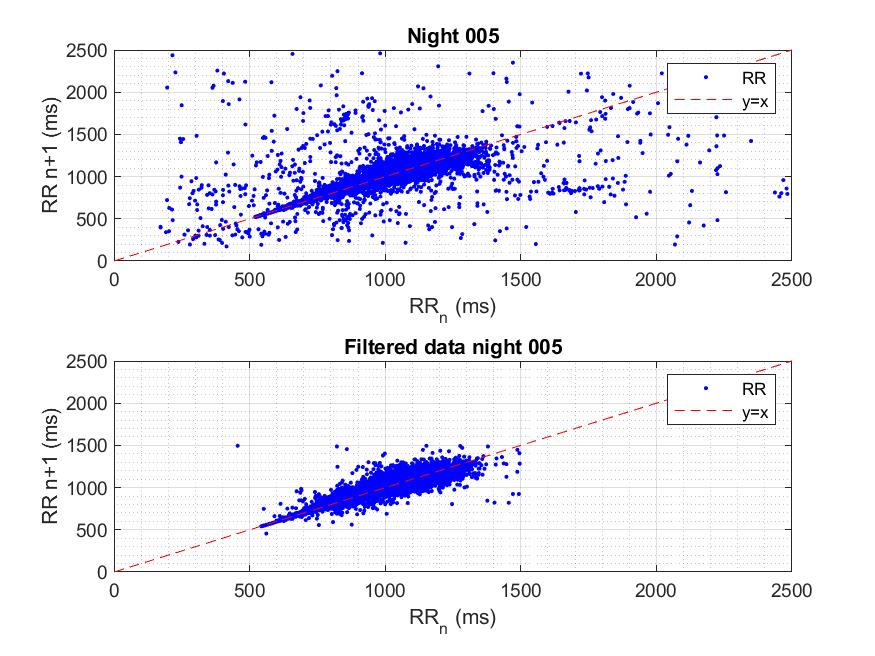

Supplement: Supplementary file 1 [file sensors-19-03367-s001.zip › Supplementary1/Night005_crr.jpg]

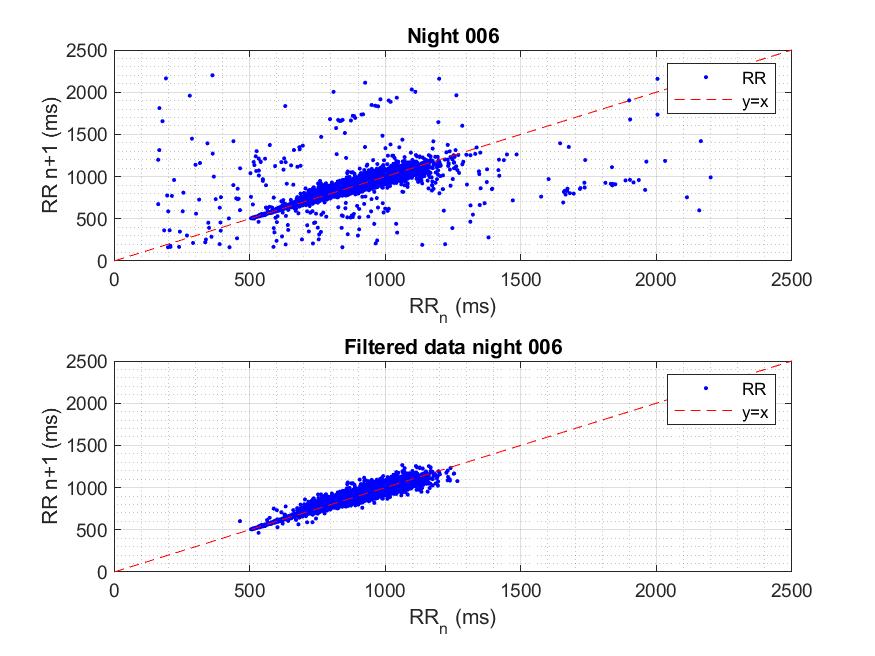

Supplement: Supplementary file 1 [file sensors-19-03367-s001.zip › Supplementary1/Night006_crr.jpg]

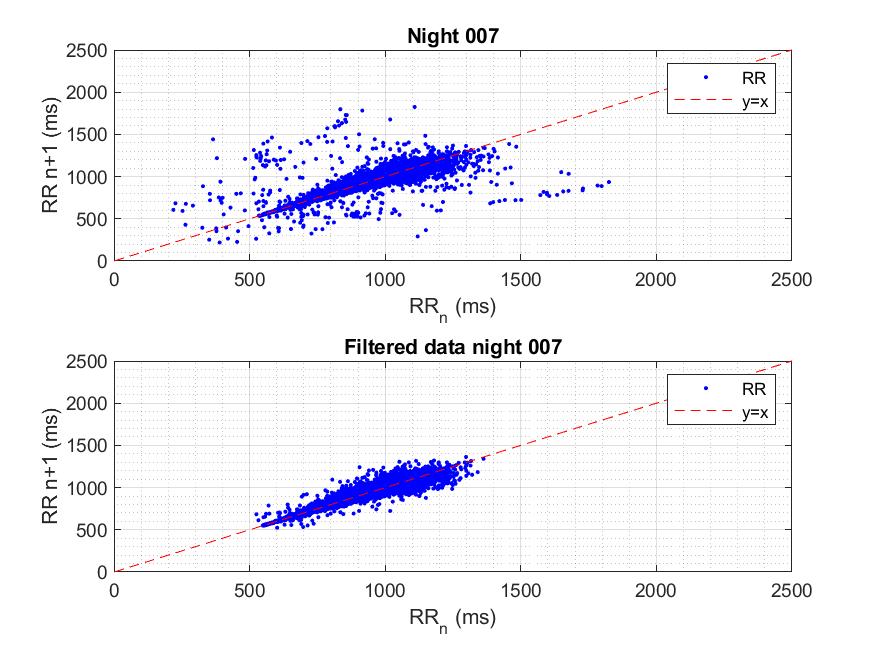

Supplement: Supplementary file 1 [file sensors-19-03367-s001.zip › Supplementary1/Night007_crr.jpg]

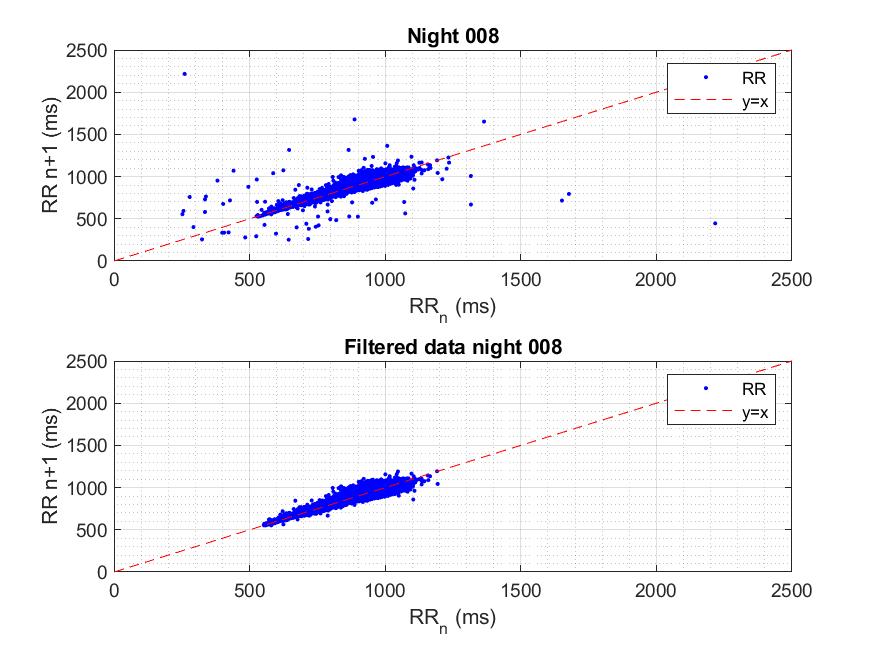

Supplement: Supplementary file 1 [file sensors-19-03367-s001.zip › Supplementary1/Night008_crr.jpg]

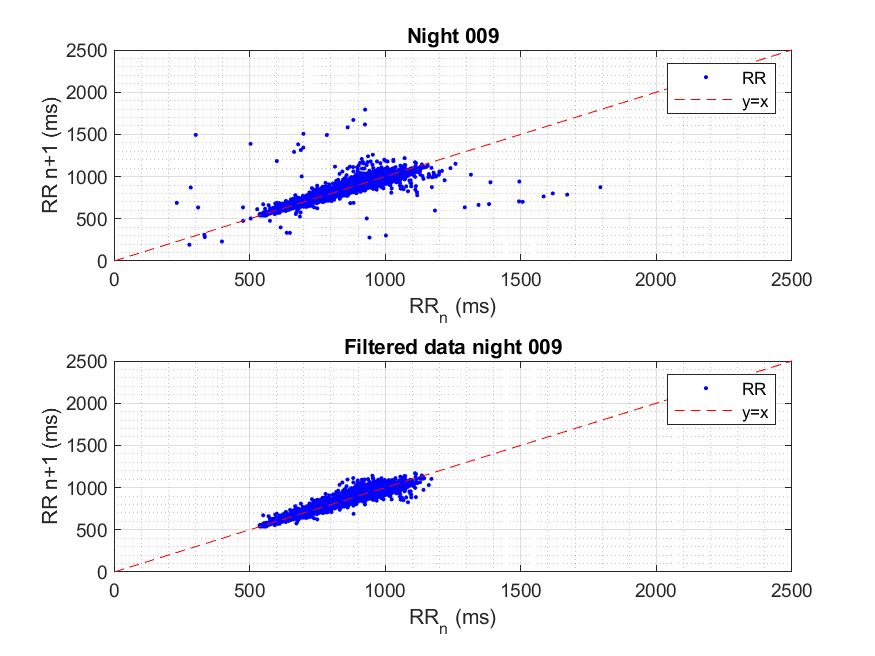

Supplement: Supplementary file 1 [file sensors-19-03367-s001.zip › Supplementary1/Night009_crr.jpg]

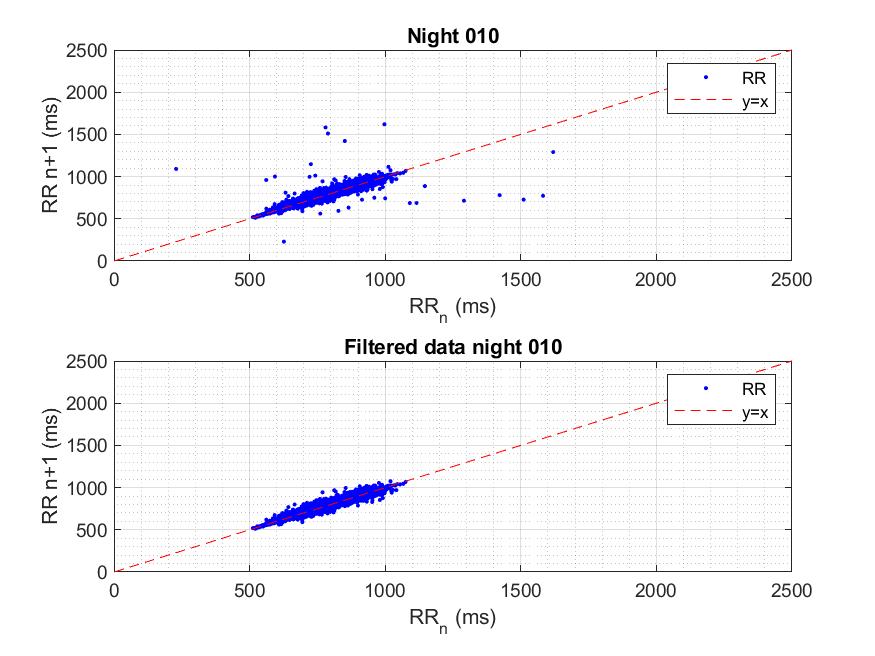

Supplement: Supplementary file 1 [file sensors-19-03367-s001.zip › Supplementary1/Night010_crr.jpg]

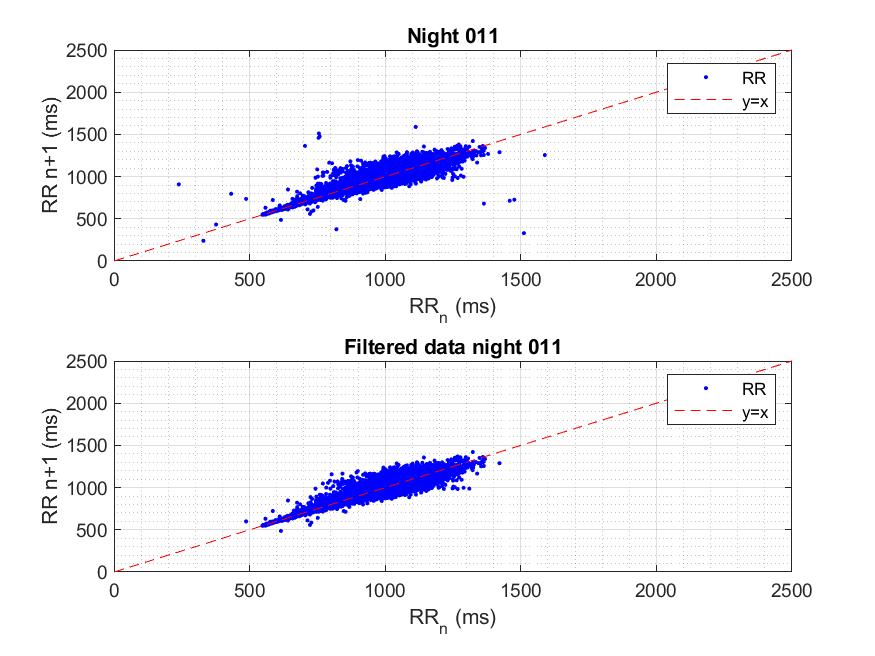

Supplement: Supplementary file 1 [file sensors-19-03367-s001.zip › Supplementary1/Night011_crr.jpg]

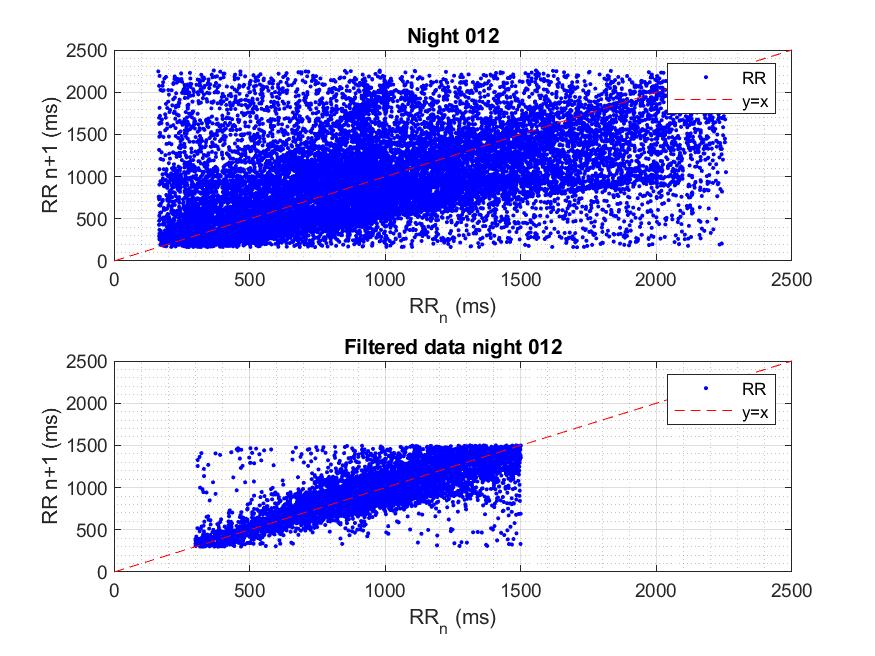

Supplement: Supplementary file 1 [file sensors-19-03367-s001.zip › Supplementary1/Night012_crr.jpg]

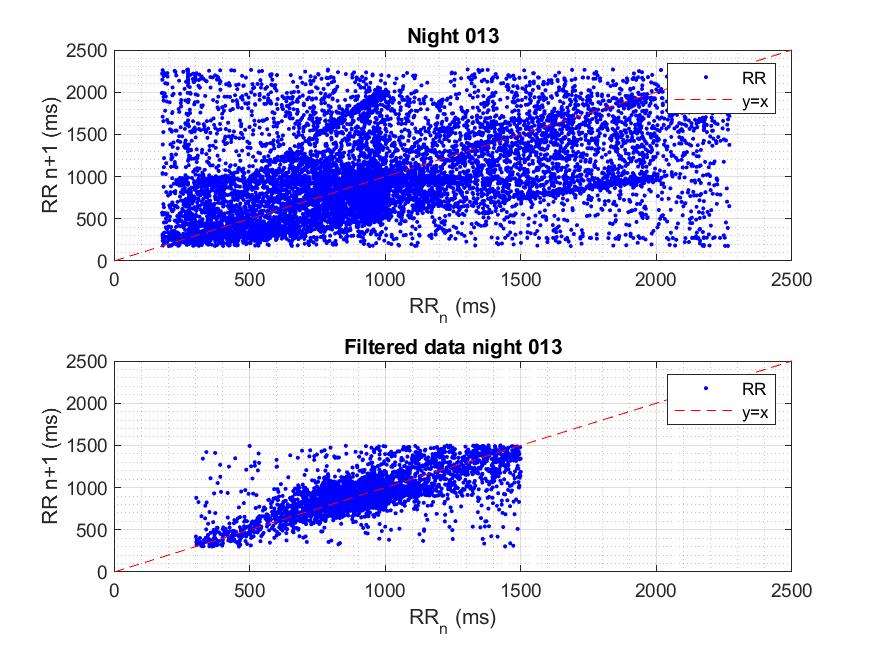

Supplement: Supplementary file 1 [file sensors-19-03367-s001.zip › Supplementary1/Night013_crr.jpg]

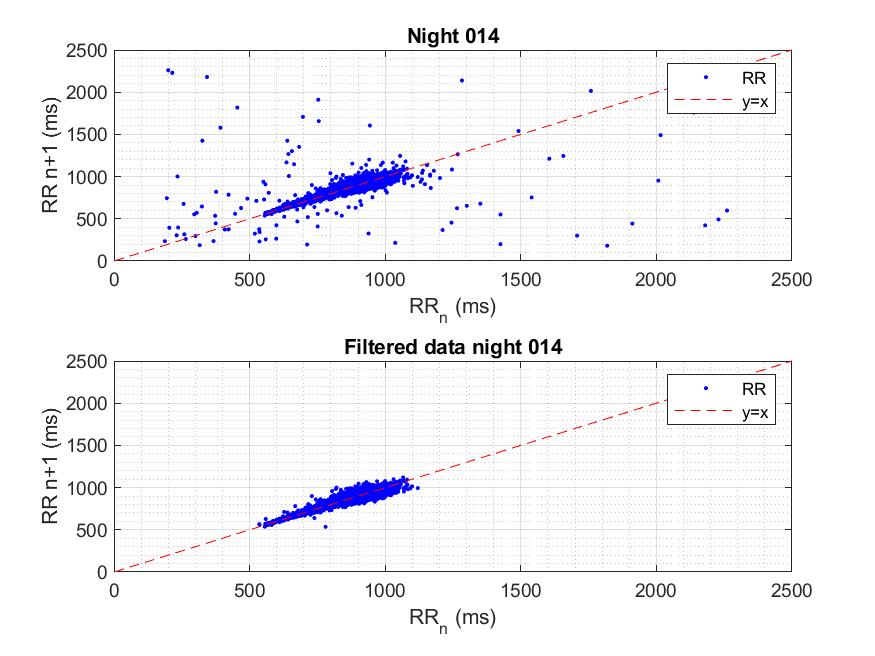

Supplement: Supplementary file 1 [file sensors-19-03367-s001.zip › Supplementary1/Night014_crr.jpg]

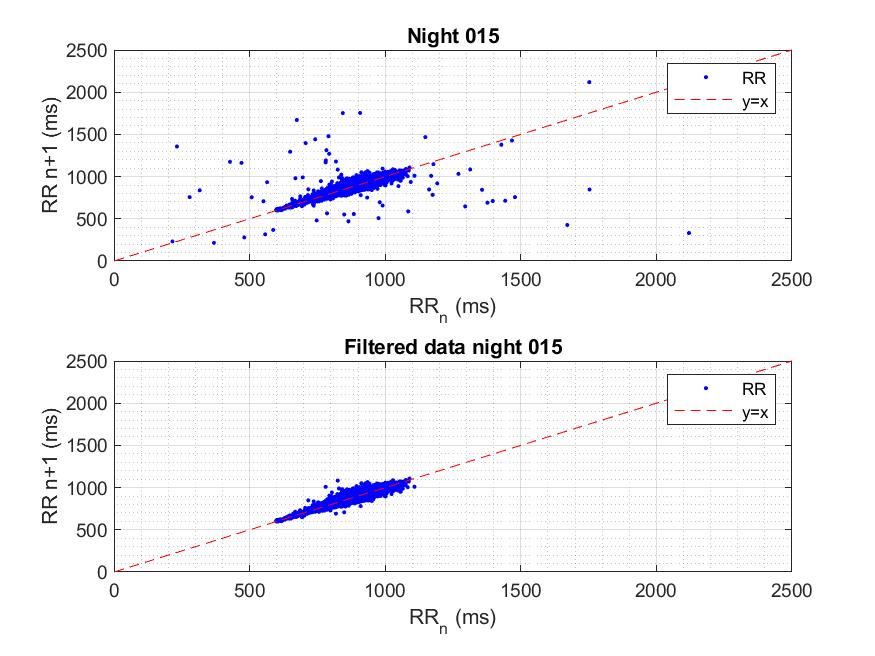

Supplement: Supplementary file 1 [file sensors-19-03367-s001.zip › Supplementary1/Night015_crr.jpg]

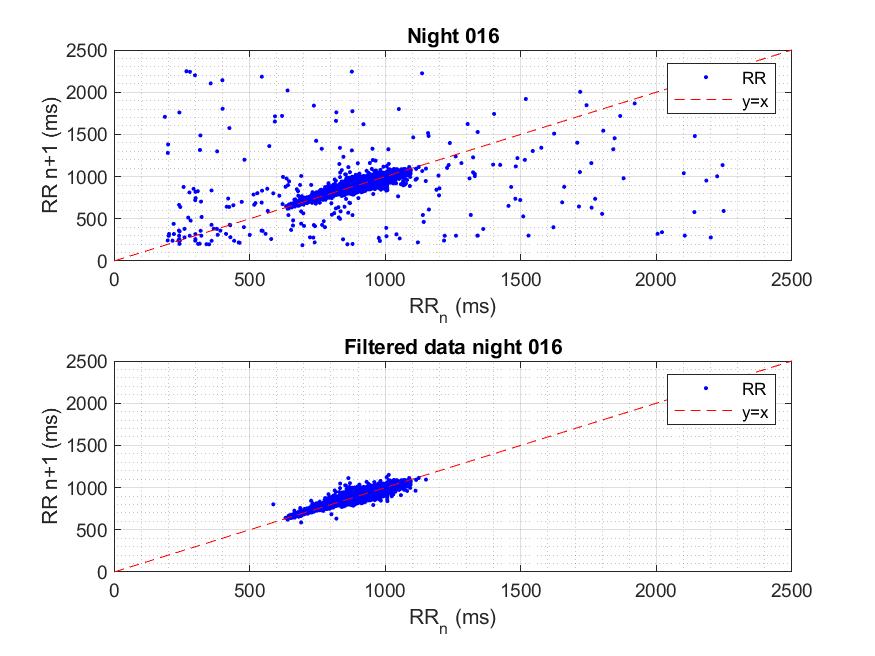

Supplement: Supplementary file 1 [file sensors-19-03367-s001.zip › Supplementary1/Night016_crr.jpg]

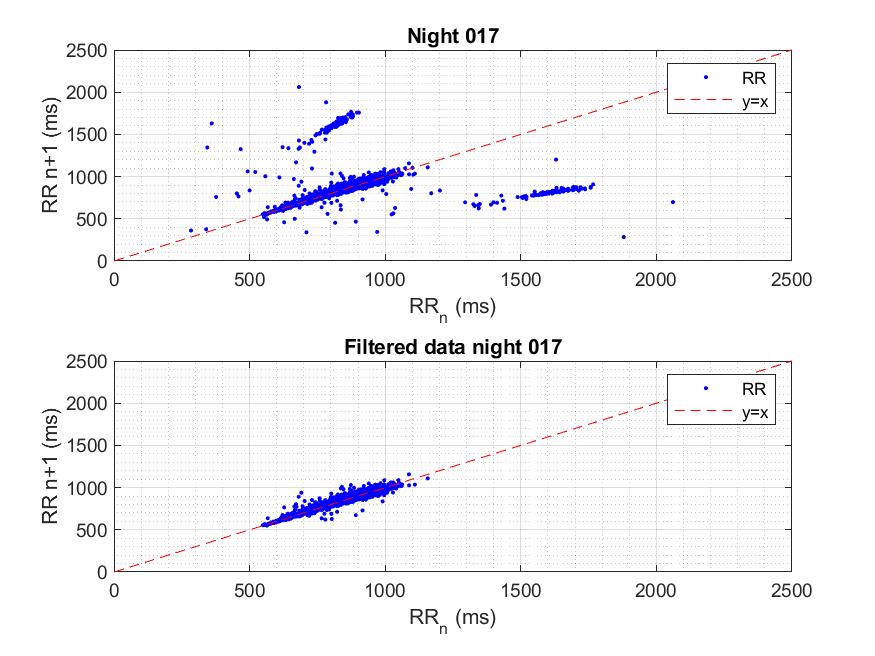

Supplement: Supplementary file 1 [file sensors-19-03367-s001.zip › Supplementary1/Night017_crr.jpg]

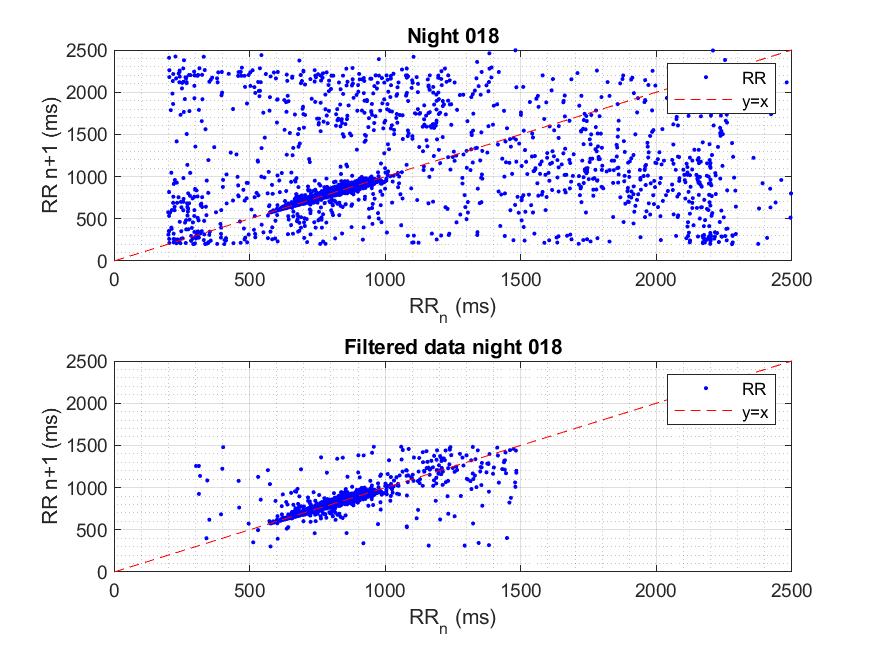

Supplement: Supplementary file 1 [file sensors-19-03367-s001.zip › Supplementary1/Night018_crr.jpg]

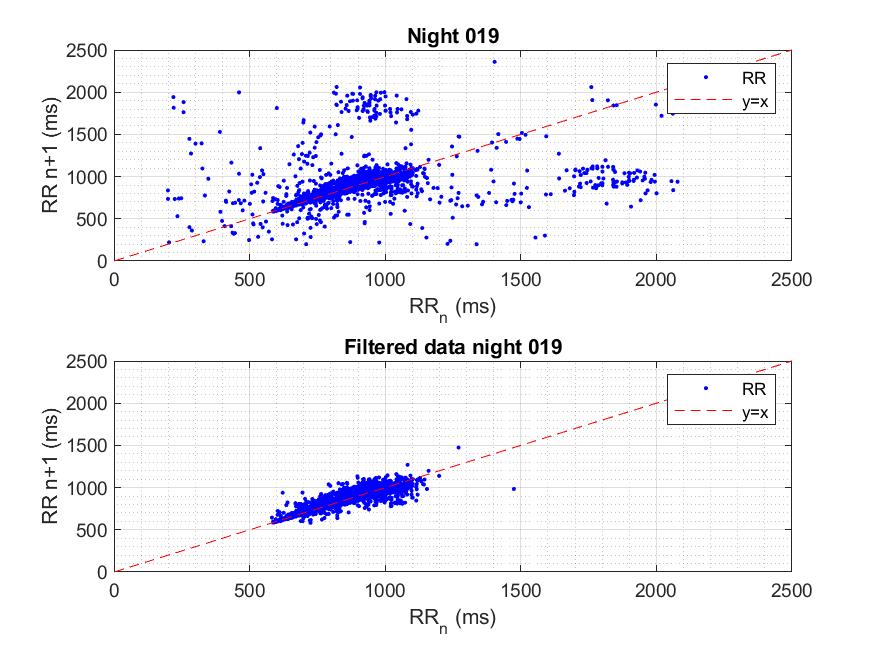

Supplement: Supplementary file 1 [file sensors-19-03367-s001.zip › Supplementary1/Night019_crr.jpg]

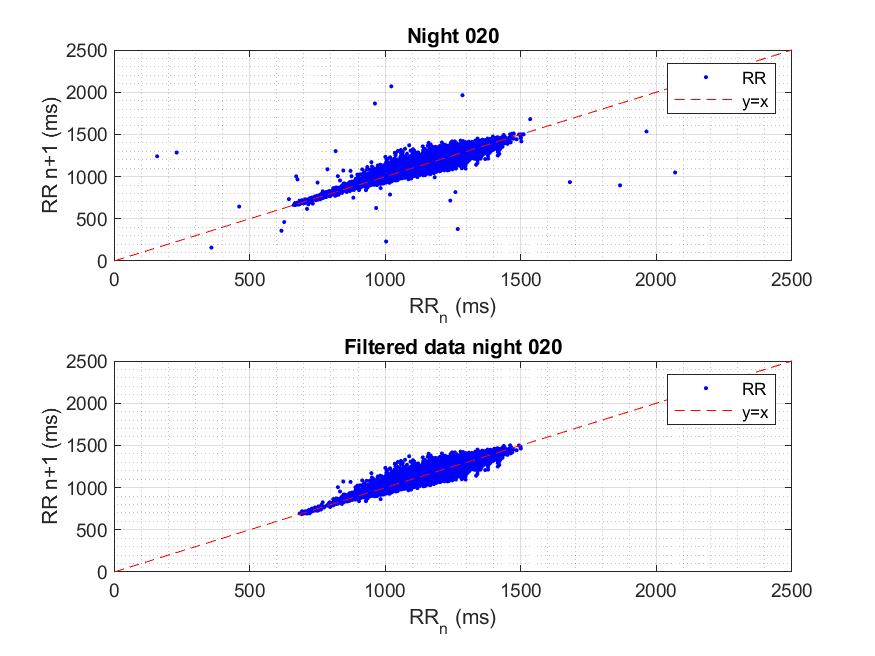

Supplement: Supplementary file 1 [file sensors-19-03367-s001.zip › Supplementary1/Night020_crr.jpg]

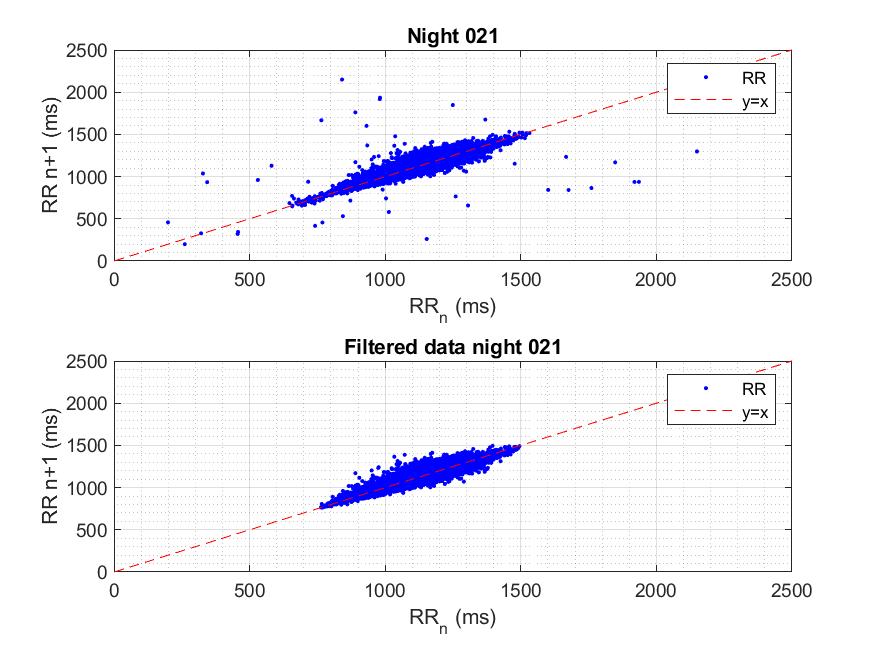

Supplement: Supplementary file 1 [file sensors-19-03367-s001.zip › Supplementary1/Night021_crr.jpg]

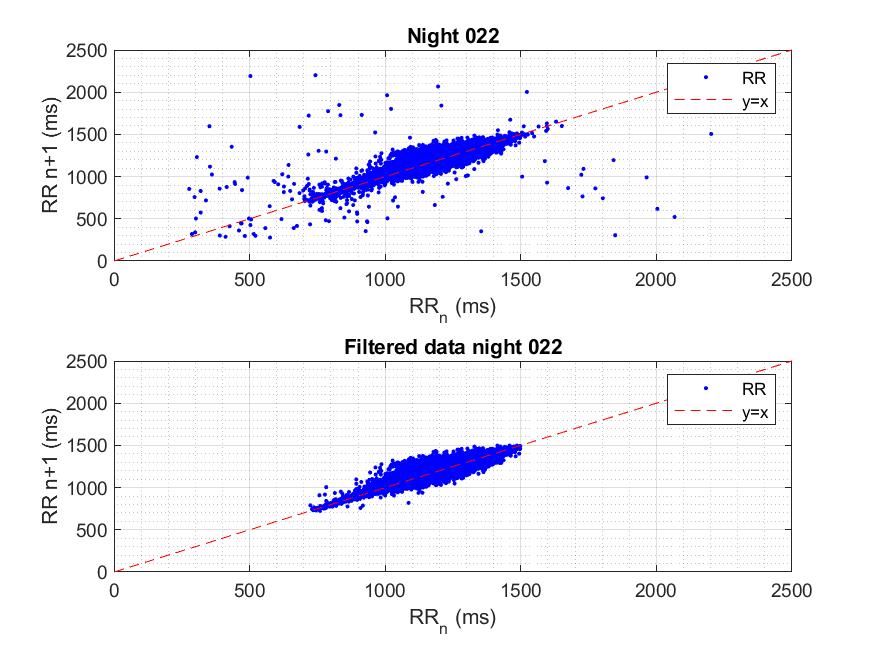

Supplement: Supplementary file 1 [file sensors-19-03367-s001.zip › Supplementary1/Night022_crr.jpg]

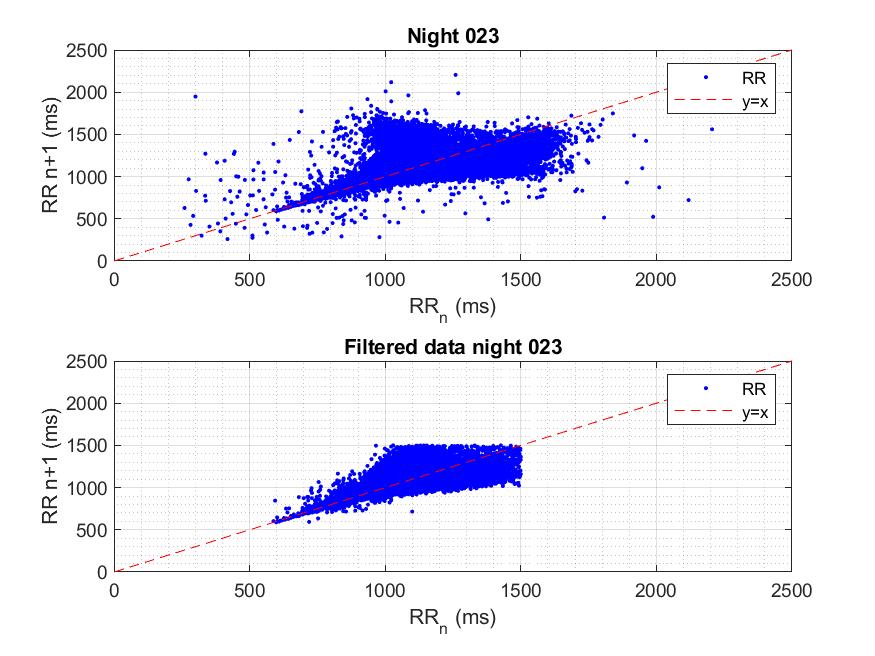

Supplement: Supplementary file 1 [file sensors-19-03367-s001.zip › Supplementary1/Night023_crr.jpg]

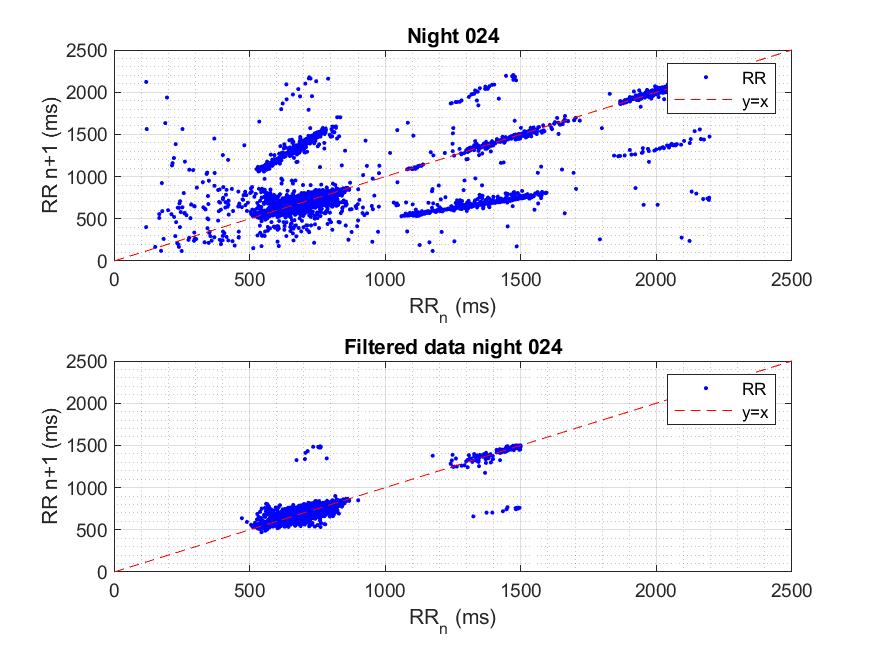

Supplement: Supplementary file 1 [file sensors-19-03367-s001.zip › Supplementary1/Night024_crr.jpg]

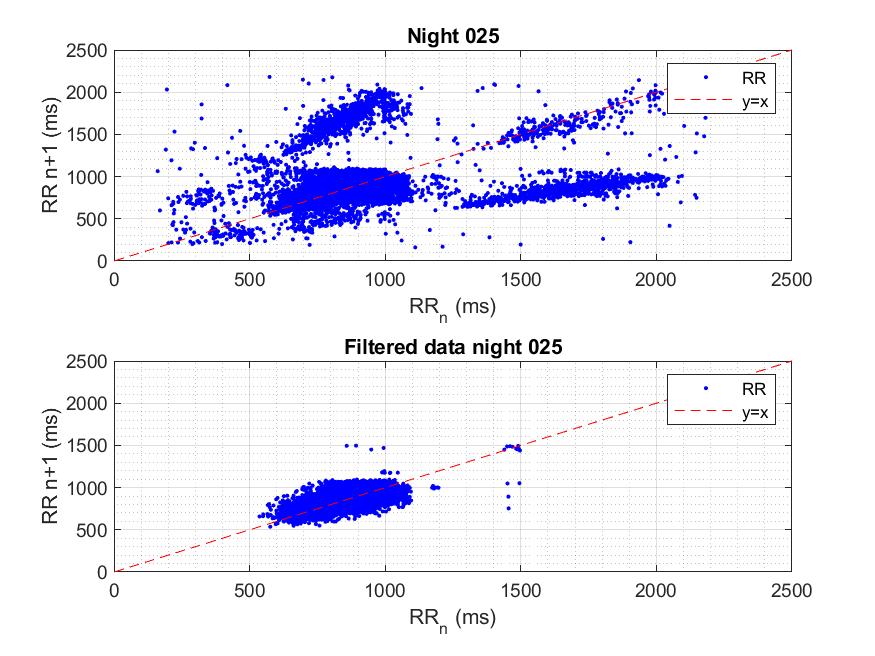

Supplement: Supplementary file 1 [file sensors-19-03367-s001.zip › Supplementary1/Night025_crr.jpg]

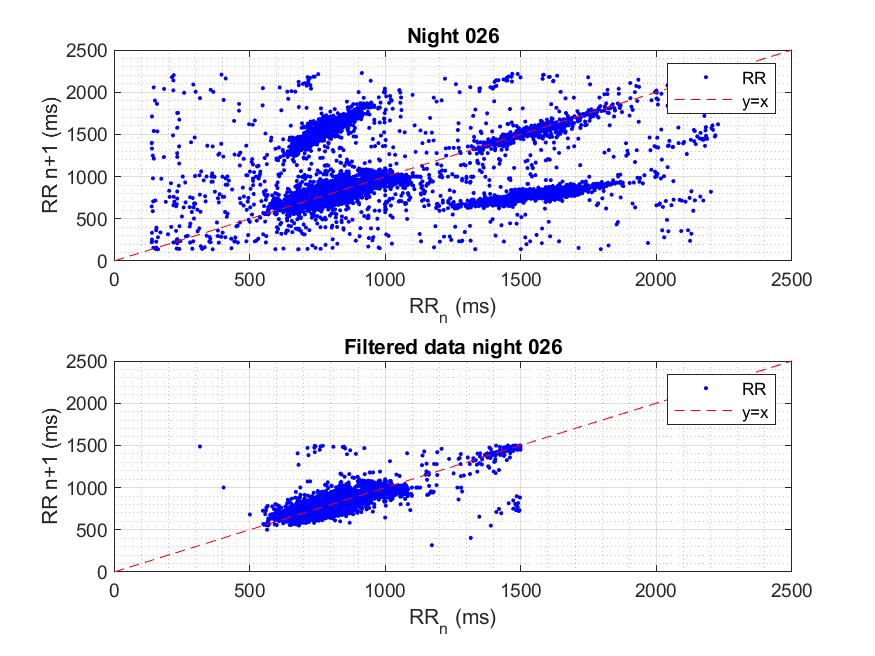

Supplement: Supplementary file 1 [file sensors-19-03367-s001.zip › Supplementary1/Night026_crr.jpg]

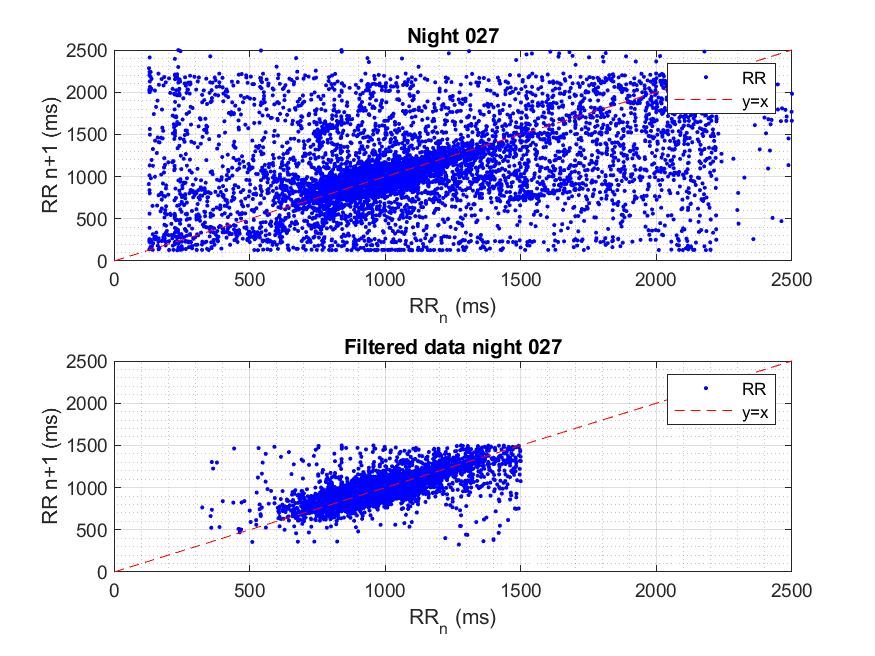

Supplement: Supplementary file 1 [file sensors-19-03367-s001.zip › Supplementary1/Night027_crr.jpg]

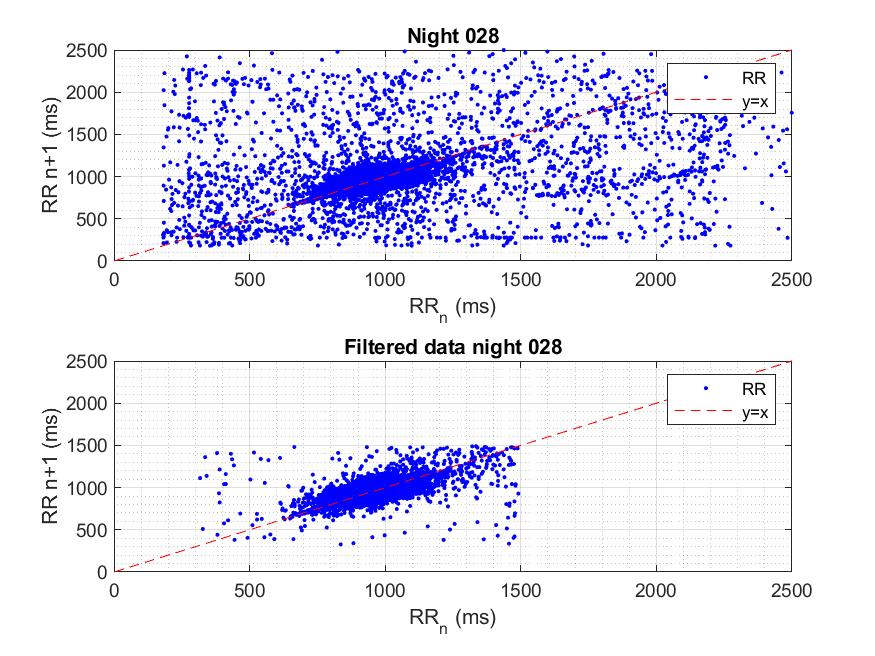

Supplement: Supplementary file 1 [file sensors-19-03367-s001.zip › Supplementary1/Night028_crr.jpg]
